# Alarmingly Occluded: When an LVAD Low-Flow Alert Signals Coronary Catastrophe

**DOI:** 10.51894/001c.165577

**Published:** 2026-07-31

**Authors:** Christian Chababi, Christopher Nedzlek, Ryan Spencer

**Affiliations:** 1 Henry Ford - Wyandotte

## 71

### Background

LVADs are increasingly encountered in emergency departments. Device alarms may reflect benign etiologies such as hypovolemia but may also signal life-threatening complications including thrombosis and myocardial infarction. The altered hemodynamics of continuous-flow devices can obscure typical presentations of acute coronary syndromes.

### Case Presentation

A 72-year-old male with advanced heart failure supported by a HeartMate III LVAD and ICD presented to the emergency department with diaphoresis, vague retrosternal chest pain, and LVAD alarm activation “low flow” hours PTP. Symptoms began during defecation. The patient reported poor oral intake following a dental procedure the previous day. During transport, EMS documented LVAD flow of 1.8 L/min and administered 500 mL intravenous crystalloid. On arrival the patient appeared pale and was bradycardic (40–50 bpm) and estimated mean arterial pressure ~75 mmHg. LVAD parameters were speed 5300 RPM, flow 3.1 L/min, PI 1.8, and power 3.5 W. Electrocardiogram demonstrated ventricular-paced bradycardia. Laboratory evaluation revealed lactate 4.6 mmol/L and rising troponin (719 → 1439 ng/L) with therapeutic anticoagulation (INR 2.75). The patient was transferred to a tertiary LVAD center where troponin exceeded assay limits (>20,000 ng/L). TTE demonstrated severe right ventricular dysfunction with concern for septal mural thrombus. CTA revealed thrombus originating in the ascending aorta below the LVAD outflow graft anastomosis with complete occlusion of the aortic lumen from the sinotubular junction to the aortic valve. The left coronary system was completely unopacified, while right coronary artery origin was also unopacified with distal reconstitution, consistent with coronary occlusion. The patient was started on dobutamine and epinephrine infusions for cardiogenic shock and anticoagulation with heparin infusion. Due to extensive myocardial injury and RV failure, the patient was listed for and subsequently underwent orthotopic heart transplantation, complicated by acute RV failure requiring TV ring placement.

### Conclusion

This case highlights aortic root thrombosis associated with LVAD outflow graft resulting in coronary artery occlusion and massive MI. Emergency physicians should maintain high suspicion for thrombotic complications in LVAD patients presenting with low-flow alarms and atypical chest pain, even when device parameters appear stable. LVAD physiology may mask ischemic presentations, making early LVAD team consultation and rapid transfer essential.

